# Prescriptions

**Published:** 1895-10

**Authors:** 


EPILEPSY.

℞. Potassi bromidi .......... ℥ j.
Sodii bromid ............. ℥ ss.
Ammonii bromid ......... ʒ ij.
Syrup ..................... f℥ ij.
Aq. gaultheriæ., q. s. ad . f℥ vj.
M. Sig.:—A teaspoonful t. d. (For a child of seven.)—*Rex.*

WHOOPING COUGH.

℞. Fl. ext. eucalypti ........ ℥ i.

Ammon. muriat.......... ʒ i.

Ext. glycyrrhizæ......... ʒ i.

Glycerini q. s ............ ℥ iv.

M. Sig.:—A teaspoonful every three or four hours.

COUGH.

℞. Codeine sulph .......... gr, xvi.

Ammon. bromid ....... ʒ v.

Aquæ. ................. ℥ ij.

Syrup ............ q. s ℥ iv.

M. Sig.:—A teaspoonful two to four times a day.

ENTERITIS.

℞. Ol. ricini ................ f ℥ j
Pulv. acaciæ ............
Sacch. alb ........... aa ℈ iss.
Tr. opii ................... m iij.
Aq. cinnam ............... ʒ xj.
M. Sig.:—Teaspoonful every four hours, for a child of one year.—*Tanner.*

DYSPEPSIA.

℞. Pepsinæ puri . . . . . . . . .gr. xxx.

Acid. hydrochlor. dil. . f ʒ ij.

Glycerini. . . . . . . . . . . . . . .f ℥ j.

Tr. gentianæ comp., q. s. ad. . . . . . . . . . . . . . . .f ʒ iij.

M. Sig. :—A teaspoonful in water, after meals. - *Aulde.*

REMITTENT FEVER.

℞. Acid. carbol. . . . . . . . . . . .f ʒ j.

Tr. iodinii comp. . . . . . .f ʒ iij.

M. Sig :—Four drops every four hours, well diluted.—*Bartholow.*

SCIATICA.

℞. Nitro-glycerine (1% alcohol solution). . . .gr. lxxv.
Tr. capsicum. . . . . . . . . .gr. cxv.
Aq. piperitæ distil. . . .gr. ccxxv
M. Sig. :—Five to ten drops, three times daily.—*Mikhaline.*

In eczema seborrhœicum of the scalp, wash the parts daily with the following solution.

℞. Sulphur precipitat. . . ℥ jss.

Camphoræ. . . . . . . . . . . . ʒ j

Mucilag. acaciæ. . . . . .f ʒ vj.

Aquæ calcis . . . . . . . . . . . .

Aquæ rosæ . . . . . . . . . . aa f ℥ xiiss.

